# Medication regimen complexity of coronary artery disease patients

**DOI:** 10.31744/einstein_journal/2021AO5565

**Published:** 2021-03-05

**Authors:** Marlon Silva Tinoco, Ronara Camila de Souza Groia-Veloso, Jéssica Nathália Domingos dos Santos, Maria Gabriela Martins Cruzeiro, Bianca Menezes Dias, Adriano Max Moreira Reis

**Affiliations:** 1 Universidade Federal de Minas Gerais Hospital das Clínicas Belo HorizonteMG Brazil Hospital das Clínicas, Universidade Federal de Minas Gerais, Belo Horizonte, MG, Brazil.; 2 Universidade Federal de Minas Gerais Faculdade de Farmácia Belo HorizonteMG Brazil Faculdade de Farmácia, Universidade Federal de Minas Gerais, Belo Horizonte, MG, Brazil.

**Keywords:** Polypharmacy, Drug utilization, Coronary artery disease, Medication adherence, Cardiovascular diseases

## Abstract

**Objective:**

To determine the factors associated with the high complexity of medication regimen in patients with coronary artery disease.

**Methods:**

A cross-sectional study was carried out in a multiprofessional cardiology outpatient clinic, in the Secondary Care of the Unified Health System, where sociodemographic (age, sex, and education), clinical (number of health conditions, cardiovascular diagnoses, and comorbidities) and pharmacotherapeutic (adherence, polypharmacy, and cardiovascular polypharmacy) characteristics were collected. These were related to complexity of medication regimen, measured through the medication regimen complexity index. The classification of high complexity of medication regimen was carried out using standardization for the older adults and stratification for adult patients, as suggested in the literature.

**Results:**

The total complexity medication regimen of 148 patients had a median of 17.0 (interquartile range of 10.5). In the univariate analysis, the factors associated with high complexity were heart failure, *diabetes mellitus*, hypertension, five or more diseases, and non-adherence to treatment. In the final model, after logistic regression, there was a statistically significant association (p<0.05) with the variables *diabetes mellitus*, hypertension, and non-adherence.

**Conclusion:**

The high complexity of medication regimen in patients with coronary artery disease was associated with the presence of *diabetes mellitus*, hypertension, and reports of non-adherence to treatment.

## INTRODUCTION

Among the chronic non-communicable diseases, cardiovascular diseases, such as coronary artery disease (CAD), are important causes of morbidity and mortality, and account for 31% of causes of death in Brazil, being the leading cause of death in the world.^(^^1^^,^^2^^)^ The pharmacological treatment of CAD involves the use of several drugs, such as antiplatelet agents, statins, beta-blockers, and angiotensin-converting enzyme inhibitors (ACEi).^(^^3^^)^ The use of multiple drugs may cause inadequate administration and a higher incidence of adverse events.^(^^4^^,^^5^^)^

Polypharmacy (use of five or more drugs), the development and availability of several drugs on the market, and epidemiological transition are factors that have contributed to the emergence of complex drug therapies.^(^^6^^)^ The medication regimen complexity is not only associated with the quantity of drugs used, but also with the dosing form, the number of doses per day, and the relation between drug use and food, among other factors.^(^^7^^-^^9^^)^

The medication regimen complexity has been associated with negative health outcomes, such as non-adherence to treatment,^(^^10^^)^ hospital readmission,^(^^11^^)^ higher risk of hospitalization,^(^^12^^)^ and mortality.^(^^13^^)^

## OBJECTIVE

To determine the factors associated with the high medication regimen complexity for patients with coronary artery disease.

## METHODS

### Sample

The convenience sample consisted of patients seen from April 2018 to February 2019 who met the selection criteria; that is, individuals diagnosed with CAD and who were using at least one drug. Patients with verbal communication difficulties were not included in the study. The patients signed an Informed Consent Form, and the study was approved by the Research Ethics Committee of the *Universidade Federal de Minas Gerais* (UFMG) (CAAE: 85804818.7.0000.5149, opinion 2585098).

### Study design and setting

This is a cross-sectional study, carried out in a multiprofessional cardiology outpatient clinic (secondary care) at the *Hospital das Clínicas da Universidade Federal de Minas Gerais* (UFMG), in Belo Horizonte (MG). It is a general hospital for medium- and high complexity cases, a reference in the care of patients of the Unified Health System (SUS - *Sistema Único de Saúde*) of the state. Patients discharged by the cardiology team are referred to this outpatient clinic and seen by a multidisciplinary team, composed of a physical therapist, clinical pharmacist, and cardiologist.

### Data collection

Data collection was performed by interviewing the patient and filling out an instrument developed for research purposes; the clinical results collected were confirmed in the medical records.

The dependent variable of the study was medication regimen complexity; the independent variables were sex, age, cardiovascular diagnosis (arrhythmias, heart failure (HF), ST-segment elevation myocardial infarction (STEMI), non-ST-segment elevation myocardial infarction (NSTEMI), unstable angina, and stable angina), comorbidities (hypertension, *diabetes mellitus*, dyslipidemia, and hypothyroidism), number of diseases, and medication regimen (polypharmacy if ≥5 drugs, excessive polypharmacy if ≥10 drugs, and cardiovascular polypharmacy if ≥5 drugs, and adhesion).

Adherence to treatment was measured by self-reporting using the 7-day Recall, which consists in measuring adherence by asking the question “In the last 7 days, on how many days have you used the drugs?” This question was asked separately for each of the medications the patient used. In measuring adherence, all medications used by patients were considered. Patients who used drugs for 6 or 7 days were classified as adherent (approximately 80% adherence), and those who used drugs for 5 days or less were classified as non-adherent.^(^^14^^)^

The medication regimen complexity was measured through the Medication Regimen Complexity Index (MRCI) validated in Brazil.^(^^15^^)^ The MRCI consists of three sections: section A, with information on dosing forms; section B, with information on dosing frequencies; and section C, with additional information. The application of MRCI was carried out by two different researchers. Disagreements between the assigned values were resolved by consensus. The MRCI score is the result of the sum of the values assigned in the three sections.

The authors responsible for validating the MRCI in Brazil authorized the use of the instrument in this research. The classification of medication regimen complexity was made using the standardization of MRCI for the elderly, in which values were considered high when above 16.5.^(^^16^^)^ The stratification suggested in the literature for adult patients considered high values as those above 13.0.^(^^17^^)^

The database was built in EpiData software, version 3.1, and the feeding was done by double data entry by different researchers.

### Data analysis

The data analysis was carried out by means of frequency results and percentage of categorical variables, and measures of central tendency and dispersion for numerical variables, with normality evaluated by the Kolmogorov-Smirnov test, considering the probability of significance with p<0.05 and 95% confidence interval.

The association between the occurrence of high medication regimen complexity and the independent variables was performed through univariate analysis, using the Pearson´s χ^2^ test. In the presence of at least one expected frequency lower than five, Fisher's exact test was used.

The independent variables that obtained a value of p≤0.20 in the univariate analysis were included in the multiple logistic regression model. In the final model, the variables that maintained a value of p<0.05 remained. In the multivariate analysis, the magnitude of the association was expressed by odds ratio (OR) with 95%CI.

To compare the models, the likelihood ratio test was used. The adequacy of the final models was evaluated by Hosmer-Lemeshow test. The statistical significance was considered when p<0.05.

The statistical analysis was performed using the SPSS software, version 25.0.

## RESULTS

A total of 148 patients participated in the study, 104 (70.3%) of whom were male. The median age was 62 years (interquartile range - IQR = 17.0).

The median number of health conditions was 5 (IQR=3.0). Among the cardiovascular diagnoses, 58.8% (n=87) of individuals had STEMI, followed by 26.4% (n=39) with NSTEMI.

Adherence to drugs was identified in 70.9% (n=105) of patients. The sociodemographic, clinical, and medication regimen characteristics are described in more detail in table 1.

**Table 1:** Sociodemographic, clinical, and medication regimen characteristics of 148 patients with coronary artery disease

Characteristics		
Age, years		62 (17.0)
Sex, male		104 (70.3)
Number of health conditions		5 (3.0)
Cardiovascular diagnoses		
	STEMI	87 (58.8)
	NSTEMI	39 (26.4)
	Arrhythmia	35 (23.7)
	Heart failure	29 (19.6)
	Unstable angina	16 (10.8)
	Stable angina	11 (7.4)
Comorbidities		
	Hypertension	107 (72.3)
	Dyslipidemia	66 (44.6)
	*Diabetes mellitus*	49 (33.1)
Adherence, as per the 7-day Recall		105 (70.9)
Polypharmacy (≥5 drugs)		135 (91.2)
Cardiovascular polypharmacy (≥5 drugs)		110 (74.3)

Results expressed as median (interquartile range) or n (%).

STEMI: ST-segment elevation myocardial infarction; NSTEMI: non-ST-segment elevation myocardial infarction.

The frequency of high medication regimen complexity was 101 patients (68.2%). The total medication regimen complexity of 148 patients presented with a median equal to 17.0 (IQR=10.5; minimum of 7.0, and maximum of 45.0), with a median of 1.0 (IQR=0.0; minimum of 1.0, and maximum of 5.0) in section A; 9.5 (IQR=19.5; minimum of 3.0, and maximum of 22.5) in section B; and 6.0 (IQR=3.0; minimum of 0.0, and maximum of 20.0) in section C of the MRCI.

In the univariate analysis, presented on table 2, the factors associated with the high medication regimen complexity with a statistically significant difference were HF, hypertension, *diabetes mellitus*, five or more diseases, and non-adherence. In a multiple logistic regression, there was a statistically significant association (p<0.05) with the variables *diabetes mellitus*, hypertension, and non-adherence.

**Tabela 2:** Uni- and multivariate analysis of factors associated with adherence to cardiovascular drugss

Description			High medication regimen complexity					
Variable			Frequency		Univariate analysis		Multivariate analysis	
			Yes n (%)	No n (%)	Odds ratio (95%CI)	p value	Odds ratio (95%CI)	p value
Sociodemographic								
Sex								
	Male		69 (68.3)	35 (74.5)	0.739 (0.340-1.610)	0.446		
	Female		32 (31.7)	12 (25.5)	1			
Elderly								
	Yes		57 (56.4)	29 (61.7)	0.804 (0.396-1.631)	0.545		
	No		44 (43.6)	18 (38.3)	1			
Cardiovascular diagnoses								
	Arrhythmia							
		Yes	15 (14.9)	6 (12.8)	1.192 (0.431-3.296)	0.735		
		No	86 (85.1)	41 (87.2)	1			
Heart failure								
	Yes		23 (22.8)	6 (12.8)	2.015 (0.760-5.341)	0.153		
	No		78 (77.2)	41 (87.2)	1			
STEMI								
	Yes		61 (60.4)	26 (55.3)	1.232 (0.612-2.480)	0.559		
	No		40 (39.6)	21 (44.7)	1			
NSTEMI								
	Yes		29 (28.7)	10 (21.3)	1.490 (0.656-3.387)	0.339		
	No		72 (71.3)	37 (78.7)	1			
Unstable angina								
	Yes		12 (11.9)	4 (8.5)	1.449 (0.442-4.758)	0.539		
	No		89 (88.1)	43 (91.5)	1			
Stable angina								
	Yes		9 (8.9)	2 (4.3)	2.201 (0.457-10.613)	0.503		
	No		92 (91.1)	45 (95.7)	1			
Comorbidities								
	Hypertension							
		Yes	81 (80.2)	26 (55.3)	3.271 (1.537-6.960)	0.002	2.339 (1.045-5.238)	0.039
		No	20 (19.8)	21 (44.7)	1			
High cholesterol								
	Yes		45 (44.6)	21 (44.7)	0.995 (0.496-1.996)	0.989		
	No		56 (55.4)	26 (55.3)	1			
*Diabetes mellitus*								
	Yes		44 (43.6)	5 (10.6)	6.484 (2.368-17.753)	0.000	6.128 (2.163-17.359)	0.001
	No		57 (56.4)	42 (89.4)	1			
Hypothyroidism								
	Yes		9 (8.9)	3 (6.4)	1.435 (0.370-5.563)	0.753		
	No		92 (91.1)	44 (93.6)	1			
Medication regimen								
	Number of diseases							
		<5 diseases	51 (50.5)	33 (70.2)	0.433 (0.207-0.904)	0.024		
		≥5 diseases	50 (49.5)	14 (29.8)	1			
Non-adherence								
	Yes		35 (34.7)	8 (17.0)	2.585 (1.090-6.134)	0.028	2.929 (1.166-7.358)	0.022
	No		66 (65.3)	39 (83.0)	1			

STEMI: ST-segment elevation myocardial infarction; NSTEMI: non-ST-segment elevation myocardial infarction; 95%CI: 95% confidence interval.

## DISCUSSION

The study showed that the high medication regimen complexity among patients with CAD presented a positive association with the presence of *diabetes mellitus*, hypertension, and a report of non-adherence to drugs. To the best of our knowledge, the investigation is a pioneer in analyzing medication regimen complexity of outpatients with CAD.

A systematic review of the literature of 35 studies identified the association between high medication regimen complexity and non-adherence to treatment. In most studies, it was identified that patients with more complex medication regimens were more likely not to adhere, and a direct association with non-adherence was also demonstrated.^(^^18^^)^ The use of several drugs, with distinct dosages, in several dosing forms, and with the need of additional information for a correct administration, may compromise treatment adherence.^(^^19^^)^

The MRCI is able to evaluate different aspects related to medication regimen complexity, such as the dosing form (section A), the dosing frequency (section B), and the additional information prescribed by the physician, to ensure the correct use of the drug, such as the need to fast, use with food, and specific times (section C), since the mere number of isolated drugs is not sufficient for evaluation.^(^^15^^,^^20^^)^ Among the three observation domains, section B is of great relevance, since dosing frequency is the factor that most contributes to high complexity, with the potential to be a possible point of change in favor of lower complexity.^(^^9^^,^^21^^)^ In the present study, this section presented the highest median. Section C items may have less impact on adherence, because they may suffer interferences inherent to the evaluation by different researchers.^(^^18^^)^ However, this bias may have been minimized, since, in the present study, the MRCI was applied by two different researchers.

Results showed that the presence of *diabetes mellitus* was significantly associated with the high medication regimen complexity. This important finding is in agreement with the literature, which shows non-adherence related to high medication regimen complexity in diabetic patients,^(^^22^^,^^23^^)^ and a higher chance of inadequate glycemic control.^(^^19^^)^ It is noteworthy that, when investigating medication regimen of CAD patients, it is important to consider the multimorbidity presented by patients with cardiovascular diseases. Multimorbidity is important because, a study that analyzed four retrospective cohorts of patients with different specific diseases, identified that most of the MRCI score was influenced by comorbidities.^(^^24^^)^ Multimorbidity may occur in CAD patients, and may explain the association between *diabetes mellitus* and high medication regimen complexity in the patients studied.

Similarly, hypertension, which was associated in this study to high medication regimen complexity, is in line with previous studies, which have demonstrated not only the prevalence of this association, but also the strong relation between high medication regimen complexity and non-adherence.^(^^25^^)^ Chronic non-communicable diseases, such as hypertension, are a great challenge to improving adherence. Patients with these diseases do not always have symptoms that help to remind of the need to use the drugs appropriately.^(^^26^^)^

Pharmacists, when performing their interventions relative to the use of medication, play an important role in increasing adherence of patients with cardiovascular diseases, since care given by this professional takes into account the singularity of the patient in terms of symptoms and beliefs about their disease and its treatment, which are important causes of non-adherence.^(^^27^^)^

The use of polypills (a term that covers solid dosing forms with a combined fixed dose of several drugs) was also a strategy detected in meta-analysis with 3,140 patients, in six countries, with a significant impact on adherence, systolic blood pressure, low-density lipoprotein cholesterol (LDL-cholesterol) in patients with cardiovascular diseases.^(^^28^^)^ The main barriers to the use of polypills are their high costs, both to the health system and to patients. Added to the fact that they are not on the list of essential drugs, the unavailability of several different doses of the same drug that compose the polypills makes it difficult to adjust the dose, which is often necessary in the management of cardiovascular diseases. However, the use of polypills in elderly patients is a strategy that can be safer,^(^^29^^)^ since they are susceptible to greater difficulty in adherence, caused by biological (such as cognitive changes and dementia, among others), psychic (depression and anxiety, for instance), and social (greater risk of socio-familial fragility) factors.^(^^30^^)^

In this study, MRCI was used to quantify the complexity of drug therapy for CAD patients. It is a tool validated in Brazil with the purpose of identifying possible justifications for non-adherence to the proposed therapy, and, consequently, for negative health outcomes. Furthermore, it is a pioneer study in evaluating medication regimen complexity in CAD outpatients.

As limitations of this study, we can mention the fact that the study was restricted to CAD patients, from a single outpatient clinic at a teaching hospital, which hinders generalizing to other patients with cardiovascular disease or other conditions, who are seen at health services of different care levels.

The results found in the study are important in determining and discussing the factors associated with high medication regimen complexity of CAD patients in the context of SUS Secondary Care. The determination of these factors assists healthcare professionals in identifying points to be addressed to minimize the negative health outcomes caused by high medication regimen complexity.

## CONCLUSION

High medication regimen complexity rates were identified. The high medication regimen complexity in patients with coronary artery disease was positively associated with the presence of *diabetes mellitus*, hypertension, and report of non-adherence to drugs.

## References

[1] 1. Ribeiro AL, Duncan BB, Brant LC, Lotufo PA, Mill JG, Barreto SM. Cardiovascular health in Brazil: trends and perspectives. Circulation. 2016;133(4):422-33. Review. 10.1161/CIRCULATIONAHA.114.008727 26811272

[2] 2. Silveira EL, Cunha LM, Pantoja MS, Lima AV, Cunha AN. Prevalência e distribuição de fatores de risco cardiovascular em portadores de doença arterial coronariana no Norte do Brasil. Rev Fac Ciênc Méd Sorocaba. 2018;20(3):167-73.

[3] 3. Sociedade Brasileira de Cardiologia (SBC). Diretrizes de doenças coronárias estável. Arq Bras Cardiol. 2014;103(Supl 2):45.

[4] 4. Payne RA, Abel GA, Avery AJ, Mercer SW, Roland MO. Is polypharmacy always hazardous? A retrospective cohort analysis using linked electronic health records from primary and secondary care. Br J Clin Pharmacol. 2014;77(6):1073-82. 10.1111/bcp.12292 PMC4093932 24428591

[5] 5. Rozenfeld S. Prevalência, fatores associados e mau uso de medicamentos entre os idosos: uma revisão. Cad Saude Publica. 2003;19(3):717-24. Review. 10.1590/s0102-311x2003000300004 12806474

[6] 6. Muir AJ, Sanders LL, Wilkinson WE, Schmader K. Reducing medication regimen complexity: a controlled trial. J Gen Intern Med. 2001;16(2):77-82. 10.1046/j.1525-1497.2001.016002077.x PMC1495168 11251757

[7] 7. George J, Phun YT, Bailey MJ, Kong DC, Stewart K. Development and validation of the medication regimen complexity index. Ann Pharmacother. 2004;38(9):1369-76. 10.1345/aph.1D479 15266038

[8] 8. Wimmer BC, Cross AJ, Jokanovic N, Wiese MD, George J, Johnell K, et al. Clinical outcomes associated with medication regimen complexity in older people: a systematic review. J Am Geriatr Soc. 2017;65(4):747-53. Review. 10.1111/jgs.14682 27991653

[9] 9. Bryant BM, Libby AM, Metz KR, Page RL 2nd, Ambardekar AV, Lindenfeld J, et al. Evaluating Patient-Level Medication Regimen Complexity Over Time in Heart Transplant Recipients. Ann Pharmacother. 2016;50(11):926-34. 10.1177/1060028016657552 27371949

[10] 10. Abada S, Clark LE, Sinha AK, Xia R, Pace-Murphy K, Flores RJ, et al. Medication regimen complexity and low adherence in older community-dwelling adults with substantiated self-neglect. J Appl Gerontol. 2019;38(6):866-83. 10.1177/0733464817714565 28645225

[11] 11. Abou-Karam N, Bradford C, Lor KB, Barnett M, Ha M, Rizos A. Medication regimen complexity and readmissions after hospitalization for heart failure, acute myocardial infarction, pneumonia, and chronic obstructive pulmonary disease. SAGE Open Med. 2016;4:2050312116632426. 10.1177/2050312116632426 PMC4778087 26985392

[12] 12. Elliott RA, O’Callaghan C, Paul E, George J. Impact of an intervention to reduce medication regimen complexity for older hospital inpatients. Int J Clin Pharm. 2013;35(2):217-24. 10.1007/s11096-012-9730-3 23212732

[13] 13. Wimmer BC, Bell JS, Fastbom J, Wiese MD, Johnell K. Medication regimen complexity and polypharmacy as factors associated with all-cause mortality in older people: a population-based cohort study. Ann Pharmacother. 2016;50(2):89-95. 10.1177/1060028015621071 PMC4714103 26681444

[14] 14. Sevilla-Cazes J, Finkleman BS, Chen J, Brensinger CM, Epstein AE, Streiff MB, et al. Association between patient-reported medication adherence and anticoagulation control. Am J Med. 2017;130(9):1092-8.e2. 10.1016/j.amjmed.2017.03.038 PMC5572106 28454906

[15] 15. Melchiors AC, Correr CJ, Fernández-Llimos F. Translation and validation into Portuguese language of the medication regimen complexity index. Arq Bras Cardiol. 2007;89(4):210-8. 10.1590/s0066-782x2007001600001 17992376

[16] 16. Pantuzza LL, Ceccato MD, Silveira MR, Pinto IV, Reis AM. Validation and standardization of the Brazilian version of the Medication Regimen Complexity Index for older adults in primary care. Geriatr Gerontol Int. 2018;18(6):853-9. 10.1111/ggi.13261 29380500

[17] 17. Ferreira JM, Galato D, Melo AC. Medication regimen complexity in adults and the elderly in a primary healthcare setting: determination of high and low complexities. Pharm Pract (Granada). 2015;13(4):659. 10.18549/PharmPract.2015.04.659 PMC4696124 26759621

[18] 18. Pantuzza LL, Ceccato MD, Silveira MR, Junqueira LM, Reis AM. Association between medication regimen complexity and pharmacotherapy adherence: a systematic review. Eur J Clin Pharmacol. 2017;73(11):1475-89. Review. 10.1007/s00228-017-2315-2 28779460

[19] 19. Advinha AM, de Oliveira-Martins S, Mateus V, Pajote SG, Lopes MJ. Medication regimen complexity in institutionalized elderly people in an aging society. Int J Clin Pharm. 2014;36(4):750-6. 10.1007/s11096-014-9963-4 24906719

[20] 20. Hirsch JD, Metz KR, Hosokawa PW, Libby AM. Validation of a patient-level medication regimen complexity index as a possible tool to identify patients for medication therapy management intervention. Pharmacotherapy. 2014; 34(8):826-35. 10.1002/phar.1452 PMC4260116 24947636

[21] 21. Elliott RA. Reducing medication regimen complexity for older patients prior to discharge from hospital: feasibility and barriers. J Clin Pharm Ther. 2012;37(6):637-42. 10.1111/j.1365-2710.2012.01356.x 22607618

[22] 22. Ayele AA, Tegegn HG, Ayele TA, Ayalew MB. Medication regimen complexity and its impact on medication adherence and glycemic control among patients with type 2 diabetes mellitus in an Ethiopian general hospital. BMJ Open Diabetes Res Care. 2019;7(1):e000685. 10.1136/bmjdrc-2019-000685 PMC6606061 31321061

[23] 23. Cramer JA. A systematic review of adherence with medications for diabetes. Diabetes Care. 2004;27(5):1218-24. Review. 10.2337/diacare.27.5.1218 15111553

[24] 24. Libby AM, Fish DN, Hosokawa PW, Linnebur SA, Metz KR, Nair KV, et al. Patient-level medication regimen complexity across populations with chronic disease. Clin Ther. 2013;35(4):385-98.e1. 10.1016/j.clinthera.2013.02.019 23541707

[25] 25. Obreli-Neto PR, Prado MF, Vieira JC, Fachini FC, Pelloso SM, Marcon SS, et al. Fatores interferentes na taxa de adesão à farmacoterapia em idosos atendidos na rede pública de saúde do Município de Salto Grande – SP, Brasil. Rev Ciênc Farm Basica Apl. 2010;31(3):229-33.

[26] 26. Nichols-English G, Poirier S. Optimizing adherence to pharmaceutical care plans. J Am Pharm Assoc (Wash). 2000;40(4):475-85. Review. 10932456

[27] 27. Hovland R, Bremer S, Frigaard C, Henjum S, Faksvåg PK, Saether EM, et al. Effect of a pharmacist-led intervention on adherence among patients with a first-time prescription for a cardiovascular medicine: a randomized controlled trial in Norwegian pharmacies. Int J Pharm Pract. 2020;28(4):337-45. 10.1111/ijpp.12598 PMC7384053 31886591

[28] 28. Webster R, Patel A, Selak V, Billot L, Bots ML, Brown A, Bullen C, Cass A, Crengle S, Raina Elley C, Grobbee DE, Neal B, Peiris D, Poulter N, Prabhakaran D, Rafter N, Stanton A, Stepien S, Thom S, Usherwood T, Wadham A, Rodgers A; SPACE Collaboration. Effectiveness of fixed dose combination medication (‘polypills’) compared with usual care in patients with cardiovascular disease or at high risk: a prospective, individual patient data meta-analysis of 3140 patients in six countries. Int J Cardiol. 2016;205:147-56. 10.1016/j.ijcard.2015.12.015 26736090

[29] 29. Salam A, Praveen D, Patel A, Tewari A, Webster R. Barriers and facilitators to the use of cardiovascular fixed-dose combination medication (Polypills) in Andhra Pradesh, India: a mixed-methods study. Glob Heart. 2019;14(3):303-10. 10.1016/j.gheart.2019.07.002 31451238

[30] 30. Moraes EN. A arte da (Des) prescrição no idoso: a dualidade terapêutica. Livro 4. Belo Horizonte: Folium Editorial; 2018. p. 34. [Coleção Guia de Bolso em Geriatria e Gerontologia].

