# Supplementary material for: Medication regimen complexity of coronary artery disease patients
Source: Einstein (Sao Paulo). 2021 Mar 5;19:eAO5565. doi: 10.31744/einstein_journal/2021AO5565 (PMC7935084; doi:10.31744/einstein_journal/2021AO5565)
Supplement: Translation

# Translation: Complexidade da farmacoterapia de pacientes com doença arterial coronariana

**Authors:** Marlon Silva Tinoco, Ronara Camila de Souza Groia-Veloso, Jéssica Nathália Domingos dos Santos, Maria Gabriela Martins Cruzeiro, Bianca Menezes Dias, Adriano Max Moreira Reis

**Affiliations:** 1 Belo HorizonteMG Brasil Hospital das Clínicas, Universidade Federal de Minas Gerais, Belo Horizonte, MG, Brasil.; 2 Belo HorizonteMG Brasil Faculdade de Farmácia, Universidade Federal de Minas Gerais, Belo Horizonte, MG, Brasil.

**Keywords:** Polimedicação, Uso de medicamentos, Doença da artéria coronariana, Adesão à medicação, Doenças cardiovasculares

## Abstract

**Objetivo:**

Determinar os fatores associados à complexidade alta da farmacoterapia em pacientes com doença arterial coronariana.

**Métodos:**

Realizou-se um estudo transversal em um ambulatório multiprofissional de cardiologia na Atenção Secundária do Sistema Único de Saúde, de onde foram coletadas características sociodemográficas (idade, sexo e escolaridade), clínicas (número de condições de saúde, diagnósticos cardiovasculares e comorbidades) e farmacoterápicas (adesão, polifarmácia e polifarmácia cardiovascular). Essas características foram relacionadas com a complexidade da farmacoterapia, mensurada por meio do Índice de Complexidade da Farmacoterapia. A classificação em complexidade alta da farmacoterapia foi realizada empregando a normatização para idosos e a estratificação para pacientes adultos, sugeridas na literatura.

**Resultados:**

A complexidade da farmacoterapia total dos 148 pacientes apresentou mediana igual a 17,0 (amplitude interquartílica de 10,5). Na análise univariada, os fatores associados à complexidade alta foram insuficiência cardíaca, *diabetes mellitus*, hipertensão arterial, cinco ou mais doenças e não adesão. No modelo final, após regressão logística, houve associação estatisticamente significante (p<0,05) com as variáveis *diabetes mellitus*, hipertensão arterial e não adesão.

**Conclusão:**

A complexidade alta da farmacoterapia em pacientes com doença arterial coronariana foi associada à presença de *diabetes mellitus*, hipertensão arterial e relato de não adesão a medicamentos

## INTRODUÇÃO

Dentre as doenças crônicas não transmissíveis, as cardiovasculares, como a doença arterial coronariana (DAC), são importantes causas de morbimortalidade, sendo responsáveis por 31% das causas de morte no Brasil e a principal causa de morte no mundo.^(^^1^^,^^2^^)^ O tratamento farmacológico da DAC envolve o uso de vários medicamentos, dentre eles antiagregantes plaquetários, estatinas, betabloqueadores e inibidores da enzima conversora de angiotensina I (iECA).^(^^3^^)^ O uso de múltiplos medicamentos pode causar inadequação em sua administração e maior incidência de eventos adversos.^(^^4^^,^^5^^)^

A polifarmácia (uso de cinco ou mais medicamentos), o desenvolvimento e a disponibilidade de diversos medicamentos no mercado e a transição epidemiológica são fatores que têm contribuído para o surgimento de terapias medicamentosas complexas.^(^^6^^)^ A complexidade da terapia medicamentosa não está associada apenas à quantidade de medicamentos utilizados, mas à forma farmacêutica, ao número de doses por dia, à relação do uso do medicamento com alimentos, dentre outros fatores.^(^^7^^-^^9^^)^

A complexidade da farmacoterapia foi associada a desfechos negativos em saúde, como não adesão ao tratamento,^(^^10^^)^ readmissão hospitalar,^(^^11^^)^ maior risco de hospitalização^(^^12^^)^ e mortalidade.^(^^13^^)^

## OBJETIVO

Determinar os fatores associados à complexidade alta da farmacoterapia de pacientes com doença arterial coronariana.

## MÉTODOS

### Amostra

A amostra de conveniência foi composta por pacientes que preencheram os critérios de seleção atendidos no período de abril de 2018 a fevereiro de 2019. Os critérios de seleção foram: indivíduos com diagnóstico de DAC e em uso de pelo menos um medicamento. Não foram incluídos no estudo pacientes com dificuldade de comunicação verbal. Os pacientes assinaram um Termo de Consentimento Livre e Esclarecido, e a pesquisa foi aprovada pelo Comitê de Ética em Pesquisa da Universidade Federal de Minas Gerais (UFMG) (CAAE: 85804818.7.0000.5149, parecer 2585098).

### Delineamento e local do estudo

Trata-se de um estudo transversal, realizado em um ambulatório multiprofissional de cardiologia da Atenção Secundária, no Hospital das Clínicas da Universidade Federal de Minas Gerais (UFMG), em Belo Horizonte (MG). O hospital é geral de média e alta complexidade, referência no atendimento de pacientes do Sistema Único de Saúde (SUS) do estado. Os pacientes que recebem alta hospitalar da equipe de cardiologia são encaminhados para esse ambulatório e atendidos por uma equipe multiprofissional, composta por fisioterapeuta, farmacêutico clínico e cardiologista.

### Coleta dos dados

A coleta de dados foi realizada por entrevista com o paciente e preenchimento de um instrumento desenvolvido para fins de pesquisa, sendo que os resultados clínicos coletados foram confirmados nos prontuários.

A variável dependente do estudo foi a complexidade da farmacoterapia e as independentes foram sexo, idade, diagnósticos cardiovasculares (arritmias, insuficiência cardíaca (IC), infarto agudo do miocárdio com supradesnivelamento do segmento ST – IAMCSST –, infarto agudo do miocárdio sem supradesnivelamento do segmento ST – IAMSSST –, angina instável e angina estável), comorbidades (hipertensão arterial, *diabetes mellitus*, dislipidemia e hipotireoidismo), número de doenças e farmacoterapia (polifarmácia se ≥5 medicamentos, polifarmácia excessiva se ≥10 medicamentos, polifarmácia cardiovascular se ≥5 medicamentos cardiovasculares e adesão).

A adesão ao tratamento foi mensurada por autorrelato utilizando o 7*-day Recall*. O 7*-day Recall* consiste na mensuração da adesão por meio da pergunta “Nos últimos 7 dias, quantos dias o senhor(a) utilizou os medicamentos?”. Essa pergunta foi feita separadamente para cada um dos medicamentos que o paciente fazia uso. Na mensuração da adesão foram considerados todos os medicamentos utilizados pelos pacientes. Os pacientes que utilizaram medicamentos por 6 ou 7 dias foram classificados como aderentes (aproximadamente 80% de adesão), e aqueles que utilizaram medicamentos por 5 dias ou menos foram classificados como não aderentes.^(^^14^^)^

A complexidade da farmacoterapia foi mensurada por meio do Índice de Complexidade da Farmacoterapia (ICFT) validado no Brasil.^(^^15^^)^ O ICFT é constituído de três seções: seção A, com informações sobre formas de dosagens; seção B, com informações de frequências de doses e seção C, que traz informações adicionais. A aplicação do ICFT foi realizada por dois pesquisadores distintos. Discordância entre os valores atribuídos foram resolvidas por consenso. O escore do IFCT é o resultado da soma dos valores atribuídos nas três seções.

Os autores responsáveis pela validação do ICFT no Brasil autorizaram o uso do instrumento nesta pesquisa. A classificação da complexidade da farmacoterapia foi realizada empregando a normatização do ICFT para idosos, sendo considerados altos valores maiores que 16,5.^(^^16^^)^ A estratificação sugerida na literatura para pacientes adultos considera como altos valores acima de 13,0.^(^^17^^)^

O banco de dados foi construído no programa EpiData, versão 3.1, e a alimentação foi realizada por dupla digitação por pesquisadores distintos.

### Análise dos dados

A análise dos dados se deu por meio dos resultados de frequência e percentagem das variáveis categóricas e medidas de tendência central e de dispersão para as variáveis numéricas, com a normalidade avaliada pelo teste de Kolmogorov-Smirnov, considerando a probabilidade de significância com p<0,05 e intervalo de confiança de 95%.

A associação entre a ocorrência de complexidade alta da farmacoterapia e as variáveis independentes foi realizada por meio de análise univariada utilizando-se o teste do χ^2^ de Pearson. Na presença de pelo menos uma frequência esperada menor que cinco, foi utilizado o teste exato de Fisher.

As variáveis independentes que obtiveram valor de p≤0,20 na análise univariada foram incluídas no modelo de regressão logística múltipla. No modelo final, permaneceram as variáveis que mantiveram valor de p<0,05. Na análise multivariada, a magnitude da associação foi expressa pela razão de chance (RC) com IC95%.

Para comparar os modelos, foi utilizado o teste de razão de verossimilhança. A adequação dos modelos finais foi avaliada pelo teste de Hosmer-Lemeshow. A significância estatística foi considerada quando p<0,05.

A análise estatística foi realizada no software SPSS, versão 25.0.

## RESULTADOS

Participaram do estudo 148 pacientes, sendo 104 (70,3%) do sexo masculino. A mediana de idade foi de 62 anos (amplitude interquartílica – IIQ – de 17,0).

A mediana do número de condições de saúde foi 5 (IIQ=3,0). Dentre os diagnósticos cardiovasculares, 58,8% (n=87) dos indivíduos apresentavam IAMCSST, seguido de 26,4% (n=39) com IAMSSST.

A adesão aos medicamentos foi identificada em 70,9% (n=105) dos pacientes. As características sociodemográficas, clínicas e farmacoterápicas estão descritas com mais detalhes na tabela 1.

**Tabela 1:** Características sociodemográficas, clínicas e farmacoterápicas de 148 pacientes com doença arterial coronariana

Características		
Idade, anos		62 (17,0)
Sexo masculino		104 (70,3)
Número de condições de saúde		5 (3,0)
Diagnósticos cardiovasculares		
	IAMCSST	87 (58,8)
	IAMSSST	39 (26,4)
	Arritmia	35 (23,7)
	Insuficiência cardíaca	29 (19,6)
	Angina instável	16 (10,8)
	Angina estável	11 (7,4)
Comorbidades		
	Hipertensão arterial sistêmica	107 (72,3)
	Dislipidemia	66 (44,6)
	*Diabetes mellitus*	49 (33,1)
Adesão segundo o 7-*day Recall*		105 (70,9)
Polifarmácia (≥5 medicamentos)		135 (91,2)
Polifarmácia cardiovascular (≥5 medicamentos)		110 (74,3)

Resultados expressos como mediana (amplitude interquartílica) ou n (%).

IAMCSST: infarto agudo do miocárdio com supradesnivelamento do segmento ST; IAMSSST: infarto agudo do miocárdio sem supradesnivelamento do segmento ST.

A frequência da complexidade alta da farmacoterapia foi de 101 pacientes (68,2%). A complexidade da farmacoterapia total dos 148 pacientes apresentou mediana igual a 17,0 (IIQ=10,5; mínimo de 7,0 e máximo de 45,0), com mediana de 1,0 (IIQ=0,0; mínimo de 1,0 e máximo de 5,0) na seção A, 9,5 (IIQ=19,5; mínimo de 3,0 e máximo de 22,5) na seção B, e 6,0 (IIQ=3,0; mínimo de 0,0 e máximo de 20,0) na seção C.

Na análise univariada, apresentada na tabela 2, os fatores associados à complexidade alta da farmacoterapia, com diferença estatisticamente significante foram: IC, hipertensão arterial, *diabetes mellitus*, cinco ou mais doenças e não adesão. Na regressão logística múltipla, verificou-se associação estatisticamente significante (p<0,05) com as variáveis *diabetes mellitus*, hipertensão arterial e não adesão.

**Tabela 2:** Análise uni e multivariada dos fatores associados com a adesão a medicamentos cardiovasculares

Descrição			Complexidade alta da farmacoterapia					
Variável			Frequência		Análise univariada		Análise multivariada	
			Sim n (%)	Não n (%)	Razão de chances (IC95%)	Valor de p	Razão de chances (IC95%)	Valor de p
Sociodemográficas								
Sexo								
	Masculino		69 (68,3)	35 (74,5)	0,739 (0,340-1,610)	0,446		
	Feminino		32 (31,7)	12 (25,5)	1			
Idoso								
	Sim		57 (56,4)	29 (61,7)	0,804 (0,396-1,631)	0,545		
	Não		44 (43,6)	18 (38,3)	1			
Diagnósticos cardiovasculares								
	Arritmia							
		Sim	15 (14,9)	6 (12,8)	1,192 (0,431-3,296)	0,735		
		Não	86 (85,1)	41 (87,2)	1			
Insuficiência cardíaca								
	Sim		23 (22,8)	6 (12,8)	2,015 (0,760-5,341)	0,153		
	Não		78 (77,2)	41 (87,2)	1			
IAMCSST								
	Sim		61 (60,4)	26 (55,3)	1,232 (0,612-2,480)	0,559		
	Não		40 (39,6)	21 (44,7)	1			
IAMSSST								
	Sim		29 (28,7)	10 (21,3)	1,490 (0,656-3,387)	0,339		
	Não		72 (71,3)	37 (78,7)	1			
Angina instável								
	Sim		12 (11,9)	4 (8,5)	1,449 (0,442-4,758)	0,539		
	Não		89 (88,1)	43 (91,5)	1			
Angina estável								
	Sim		9 (8,9)	2 (4,3)	2,201 (0,457-10,613)	0,503		
	Não		92 (91,1)	45 (95,7)	1			
Comorbidades								
	Hipertensão arterial							
		Sim	81 (80,2)	26 (55,3)	3,271 (1,537-6,960)	0,002	2,339 (1,045-5,238)	0,039
		Não	20 (19,8)	21 (44,7)	1			
Colesterol alto								
	Sim		45 (44,6)	21 (44,7)	0,995 (0,496-1,996)	0,989		
	Não		56 (55,4)	26 (55,3)	1			
*Diabetes mellitus*								
	Sim		44 (43,6)	5 (10,6)	6,484 (2,368-17,753)	0,000	6,128 (2,163-17,359)	0,001
	Não		57 (56,4)	42 (89,4)	1			
Hipotireoidismo								
	Sim		9 (8,9)	3 (6,4)	1,435 (0,370-5,563)	0,753		
	Não		92 (91,1)	44 (93,6)	1			
Farmacoterápicas								
	Número de doenças							
		<5 doenças	51 (50,5)	33 (70,2)	0,433 (0,207-0,904)	0,024		
		≥5 doenças	50 (49,5)	14 (29,8)	1			
Não adesão								
	Sim		35 (34,7)	8 (17,0)	2,585 (1,090-6,134)	0,028	2,929 (1,166-7,358)	0,022
	Não		66 (65,3)	39 (83,0)	1			

IAMCSST: infarto agudo do miocárdio com supradesnivelamento do segmento ST; IAMSSST: infarto agudo do miocárdio sem supradesnivelamento do segmento ST; IC95%: intervalo de confiança de 95%.

## DISCUSSÃO

O estudo mostrou que a complexidade alta da farmacoterapia entre pacientes com DAC apresentou associação positiva com presença de *diabetes mellitus*, hipertensão arterial e relato de não adesão a medicamentos. Ao melhor do nosso conhecimento a investigação é pioneira ao analisar a complexidade da farmacoterapia de pacientes ambulatoriais com DAC.

Uma revisão sistemática da literatura de 35 estudos identificou a associação entre complexidade alta da farmacoterapia e não adesão ao tratamento. Na maioria dos estudos, foi identificado que pacientes com esquemas farmacoterápicos mais complexos eram mais propensos a não aderir, sendo demonstrada também associação direta com a não adesão.^(^^18^^)^ O uso de vários medicamentos, com posologias distintas, em diversas formas farmacêuticas e com necessidade de informações adicionais para uma administração correta, pode comprometer a adesão ao tratamento.^(^^19^^)^

O ICFT é capaz de avaliar diferentes aspectos relacionados à complexidade da farmacoterapia, como a forma farmacêutica (seção A), a frequência de administração (seção B) e as informações adicionais prescritas pelo médico para assegurar o uso correto do medicamento, como necessidade de jejum, uso com alimentos, e horários específicos (seção C), uma vez que apenas o número de medicamentos não é, de maneira isolada, suficiente para a avaliação.^(^^15^^,^^20^^)^ Dentre os três domínios de observação, a seção B é de grande relevância, pois a frequência de administração é o fator que mais contribui para uma complexidade alta, com potencial para ser um possível ponto de mudanças em prol de uma menor complexidade.^(^^9^^,^^21^^)^ Essa seção, no presente estudo, foi a que apresentou maior mediana. Os itens da seção C podem ter menos impacto na adesão, uma vez que podem sofrer interferências inerentes à avaliação pelos diferentes pesquisadores.^(^^18^^)^ Entretanto, esse viés pode ter sido minimizado uma vez que, no presente estudo, o ICFT foi aplicado por dois pesquisadores distintos.

Verificou-se que a presença de *diabetes mellitus* está significativamente associada à complexidade alta da farmacoterapia. Esse importante achado está em consonância com a literatura, que mostra a não adesão relacionada à complexidade alta da farmacoterapia em pacientes diabéticos^(^^22^^,^^23^^)^ e a uma maior chance de controle glicêmico inadequado.^(^^19^^)^ Vale ressaltar que, ao investigar a farmacoterapia do paciente com DAC, é importante considerar a multimorbidade que os pacientes com doenças cardiovasculares apresentam. A multimorbidade é importante, porque, em estudo que analisou quatro coortes retrospectivas de pacientes com diferentes doenças específicas, identificou-se que a maior parte da pontuação do ICFT foi influenciada pelas comorbidades.^(^^24^^)^ A multimorbidade, que pode ocorrer em pacientes com DAC, pode explicar a associação entre *diabetes mellitus* e complexidade alta da farmacoterapia nos pacientes estudados.

Da mesma forma, a hipertensão arterial, que foi associada neste estudo à complexidade alta da farmacoterapia, está em consonância com estudos prévios, que têm demonstrado não só a prevalência dessa associação, como também a forte relação entre a complexidade alta da farmacoterapia e a não adesão.^(^^25^^)^ As doenças crônicas não transmissíveis, como hipertensão arterial, são um grande desafio para melhoria da adesão. Os pacientes com essas doenças nem sempre têm sintomas que ajudam a lembrar a necessidade de utilizar os medicamentos adequadamente.^(^^26^^)^

O profissional farmacêutico, ao realizar suas intervenções em relação à utilização de medicamentos, desempenha papel importante no aumento da adesão de pacientes com doenças cardiovasculares, uma vez que o atendimento desse profissional leva em conta a singularidade do paciente quanto aos sintomas e às crenças sobre sua doença e seu tratamento – importantes causas da não adesão.^(^^27^^)^

O uso de polipílulas (termo que abrange formas farmacêuticas sólidas com dose fixa combinada de vários fármacos) foi também estratégia detectada em metanálise com 3.140 pacientes, em seis países, com impacto significativo na adesão, pressão arterial sistólica e lipoproteína de baixa densidade-colesterol (LDL-colesterol) em pacientes com doenças cardiovasculares.^(^^28^^)^ As principais barreiras para uso das polipílulas são seu alto custo, tanto para o sistema de saúde quanto para o próprio paciente, a indisponibilidade de várias doses diferentes de um mesmo medicamento que compõem as polipílulas, dificultando o ajuste de dose frequentemente necessário no manejo de doenças cardiovasculares, e o fato de não constarem na relação de medicamentos essenciais. Entretanto, o uso de polipílulas em pacientes idosos é uma estratégia que pode ser mais segura,^(^^29^^)^ uma vez que eles estão suscetíveis a uma maior dificuldade de adesão, causada por fatores biológicos (como alterações cognitivas e demências, entre outras), psíquicos (depressão e ansiedade, entre outras) e sociais (maior risco de fragilidade sociofamiliar).^(^^30^^)^

Neste estudo, o ICFT foi utilizado para quantificar a complexidade da terapia medicamentosa dos pacientes com DAC. Trata-se de instrumento validado no país com a finalidade de identificar possíveis justificativas para a não adesão à terapia proposta e, consequentemente, para desfechos negativos em saúde. Além disso, esse estudo é pioneiro, ao avaliar a complexidade da farmacoterapia em pacientes ambulatoriais com DAC.

Como limitações deste estudo, podemos citar o fato de o estudo ter sido restrito a pacientes com DAC de um único ambulatório de um hospital de ensino, o que dificulta a generalização para outros pacientes com doença cardiovascular ou outras patologias atendidos em serviços de saúde de diferentes níveis de complexidade assistencial.

Os resultados encontrados no estudo são importantes, ao determinarem e discutirem os fatores associados à complexidade alta da farmacoterapia de pacientes com DAC no contexto da Atenção Secundária do SUS. A determinação desses fatores auxilia os profissionais de saúde na identificação de pontos a serem abordados para minimização dos resultados negativos em saúde causados pela complexidade alta da farmacoterapia.

## CONCLUSÃO

Foram identificadas altas taxas de complexidade da farmacoterapia. A complexidade alta da farmacoterapia em pacientes com doença arterial coronariana foi associada positivamente à presença de *diabetes mellitus*, hipertensão arterial e relato de não adesão a medicamentos.
